# Effect of antinuclear antibody positivity on antineutrophil cytoplasmic antibody results by indirect immunofluorescence assay

**DOI:** 10.1097/MD.0000000000037384

**Published:** 2024-03-08

**Authors:** Demet Gür Vural, Büşra Usta, Yeliz Tanriverdi Çayci, Kemal Bilgin, Asuman Birinci

**Affiliations:** a Department of Medical Microbiology, Ondokuz Mayis University, Medical School, Samsun, Turkey.

**Keywords:** ANCA-associated vasculitis (AAV), antineutrophil cytoplasmic antibodies, antinuclear antibodies, indirect-immunofluorescence assay, interference

## Abstract

**Background:**

The indirect immunofluorescence assay (IIFA) utilizing antineutrophil cytoplasmic antibodies (ANCA) is widely used as a diagnostic test for autoimmune vasculitis. The presence of antinuclear antibodies (ANA) might lead to a misleading interpretation of ANCA. This study aims to explore the impact of the presence of ANA on the interpretation of ANCA.

**Methods:**

This retrospective research examined samples negative for antiMPO and antiPR3 ANCA by IIFA and explored correlations between the ANA–IIFA results and the ANCA interpretation frequencies. Our analysis involved the use of suitable statistical methods, including Chi-square and kappa statistics.

**Results:**

Up to 75.2% of the ANCA–IIFA-positive samples exhibited a positive p-ANCA pattern when using the ethanol-fixed substrate, with c-ANCA positivity at 24.8%. In the ANA–IIFA-positive samples, ~77.3% displayed p-ANCA patterns on ethanol-fixed substrates. A comparison between the ANA–IIFA titers and the p-ANCA results revealed that p-ANCA positivity was notably more common in samples with higher titers, and this correlation was found to be statistically significant.

**Conclusion:**

Positive ANA results by IIFA tests are linked to a higher incidence of p-ANCA interpretation, particularly in cases with higher titer patterns. This insight aids laboratories in establishing effective workflows to investigate potential p-ANCA interference.

## 1. Introduction

Systemic autoimmune diseases refer to a group of diseases that share common clinical, immunological, and genetic characteristics while possessing disease-specific markers. These diseases are developed by genetically susceptible individuals under environmental triggers. Antineutrophil cytoplasmic antibody (ANCA)-associated vasculitis presents autoimmune systemic diseases that can lead to severe morbidity and mortality due to diverse systemic manifestations.^[1]^ Globally, the yearly incidence of ANCA-associated vasculitis (AAV) varies between 10 and 20 cases per million, with mortality rates reaching up to 80%.^[2–4]^

The diagnosis of AAV is based on the combined evaluation of clinical symptoms, positive ANCA serology, and/or histological evidence of otherwise unexplained necrotizing vasculitis and/or granulomatous destructive parenchymal inflammation.^[5–7]^ ANCAs are considered one of the most important serological biomarkers for AAV.^[6]^ These antibodies react against cytoplasmic antigens expressed in the primary granules of neutrophils and the lysosomes of monocytes, particularly against neutrophil cytoplasmic components, such as proteinase 3 (PR3) and myeloperoxidase (MPO).^[8]^

The identification of ANCA is primarily based on two different methods. The first method includes indirect immunofluorescence assay (IIFA), in which specially coated slides containing fixed human leukocytes are mixed with human serum and incubated. Under an immunofluorescence microscope, the resulting patterns are reported as cytoplasmic ANCA (c-ANCA), perinuclear ANCA (p-ANCA), and atypical ANCA.^[4]^ The second method is enzyme-linked immunosorbent assay (ELISA), which is used to investigate the antibodies in a patient’s serum using PR3 and MPO antigens.^[4,8]^ Several assays other than IIF or ELISA are commercially available for the detection of MPO and PR3. For instance, dot-blot assays are highly sensitive and specific in detecting MPO and PR3 in patients with suspected AAV. In this test, when a patient’s serum is added and an incubation process is carried out, the evaluation of band intensity allows for the detection of PR3 and MPO antibodies.^[9]^

IIFA-based ANCA detection tests also have some challenges. The proteins PR3 and MPO are located within the primary granules of neutrophils and monocytes, and these proteins become visible on the cell surface due to various inflammatory triggers.^[10]^ Ethanol treatment dissolves granule membranes, facilitating the movement of these proteins. PR3 and MPO exhibit different behaviors due to their distinct isoelectric pH. For instance, highly cationic MPO relocates towards oppositely charged nuclear content, resulting in a perinuclear appearance.^[11]^

Antinuclear antibodies (ANA) recognize nuclear antigens, causing some ANAs to produce a p-ANCA pattern reminiscent of the antiMPO antibody. The distinction between a true p-ANCA and ANA is crucial, as these autoantibodies are associated with different diseases, each having distinct pathogenic mechanisms, clinical presentations, and treatment strategies.^[12]^

In this study, we aimed to investigate the impact of ANA on ANCA testing via IIFA and the associated risks of false-positive results.

## 2. Materials and methods

Samples collected for ANA (by IIFA) and ANCA (via both IIFA and ELISA) were included in this study. These samples were continuously collected for testing across various departments of our tertiary teaching hospital. Data were obtained retrospectively from January 2017 to December 2022. ANCA–IIFA positivity was the starting point of our analysis, followed by the results of the ANCA. Following the manufacturer’s recommendations (Euroimmun AG, Lübeck, Germany), sera were diluted to 1:10 and evaluated by the IIFA technique for p-ANCA and c-ANCA. Approximately 3 different areas were evaluated for each sample on these slides, consisting of Hep-2 cells, ethanol-fixed human granulocytes, and formalin-fixed human granulocytes in the solid phase. Patient sera, diluted to a ratio of 1:100, were used to detect ANA using an IIFA-based kit from Euroimmun in Germany.

PR3 and MPO were detected by ELISA using an automated Alegria system (ORGENTEC, Mainz, Germany). Our analysis involved the use of suitable statistical methods, including Chi-square and kappa statistics.

This study was conducted in accordance with the principles of the Helsinki Declaration (2013 revised edition) and received approval from the Ondokuz Mayis University Clinical Research Ethics Committee.

## 3. Results

From 2017 to 2022, a comprehensive study was conducted involving 3991 patients who underwent ANCA testing. The investigation focused on analyzing the ANA test results of 307 patients who tested positive for ANCA by IIFA but showed negative results in ELISA–MPO and ELISA–PR3.

When analyzing the demographic data of these patients, it was found that the average age was 41 years (range: 1–89; Q1: 16, Q3: 61 years), with a gender distribution of 115 (37.5%) males and 192 (62.5%) females.

Among the department admissions to the hospital, rheumatology placed first with 158 (51.5%), followed by neurology with 49 (16%), chest diseases with 31 (10.1%), nephrology with 19 (6.2%) and other departments with 50 (16.2%).

In the examination of the ANCA–IIFA results for the 307 patients, 231 (75.2%) showed p-ANCA positivity, while 76 (24.8%) showed c-ANCA positivity. Regarding the analysis of the ANA results, 86 patients (28%) were identified as negative, while 221 patients (71.9%) showed positive ANA results with various patterns (Table 1). Notably, the positive ANA results were ~2.5 times higher (221/86) compared with the ANA-negative samples with positive ANCA results. In the ANA–IIFA-positive samples, ~77.3% displayed p-ANCA patterns on ethanol-fixed substrates, whereas 26.7% were c-ANCA-positive.

**Table 1:** Results of ANA–IIFA.

ANA patterns	Count	Percentage
Granular	87	28.3%
Negative	86	28%
Granular and homogeneous	56	18.2%
Nucleolar	29	9.4%
Homogeneous	26	8.5%
GGK	8	2.6%
Granular and nucleolar	8	2.6%
Nuclear membrane	7	2.3%
Total	307	100%

The relationship between specific patterns in the ANA test and the ANCA–IIFA was examined, revealing that 74 patients showed granular cytoplasmic staining. Among these patients, 60 (81%) tested positive for p-ANCA, while 14 (18.9%) showed positivity for c-ANCA. In particular, all 14 patients with c-ANCA positivity were found to be formalin-resistant, whereas among the 60 patients with p-ANCA positivity, 14 (23%) were formalin-resistant and 46 (77%) were formalin-sensitive. The analysis of the association between cytoplasmic staining and p-ANCA positivity did not yield any statistical significance (*P* = .1).

The distribution of patterns in Table 2 shows that the homogeneous and GGK staining patterns correlate only with p-ANCA positivity. When considering the other patterns as a separate group and analyzing the association between these 2 patterns and p-ANCA positivity using the Chi-square test, a statistically significant relationship was found (*P* = .002).

**Table 2:** Distribution of ANA–IIFA results in c-ANCA/p-ANCA.

ANA patterns	c-ANCA positive	p-ANCA positive
Granular	23 (30%)	64 (28%)
Negative	26 (34%)	60 (26%)
Granular and homogeneous	6 (8%)	50 (22%)
Nucleolar	15 (20%)	14 (6%)
Homogeneous	0	26 (11%)
GGK	0	8 (3.5%)
Granular and nucleolar	4 (5%)	4 (2%)
Nuclear membrane	2 (3%)	5 (2%)
Total	76	231

Among the 86 patients with negative ANA tests, p-ANCA positivity was observed in 70% (*n* = 60), while c-ANCA positivity was observed in 30% (*n* = 26). Within the subset of patients with no nuclear pattern detected, 10 exhibited cytoplasmic granular staining. The presence of granular staining in the cytoplasm raises the possibility of a false-positive ANCA result. However, when analyzed with the Chi-square test, no statistically significant association was found (*P* = .18).

When titers from the ANA–IIFA test were compared with the p-ANCA results, it was observed that p-ANCA positivity was more prevalent in high-titer results (Table 3), and this association was found to be statistically significant (*P* = .002).

**Table 3:** Relationship between ANA titers and p-ANCA positivity.

ANA–IIFA titre	P-ANCA positive (*n*)/ANA positive (*n*)	Percentage
+1	83/123	(67.5%)
+2	47/53	(88.7%)
+3	30/34	(88.2%)
+4	11/11	(100%)

## 4. Discussion

Detection of ANCA is important in many inflammatory diseases that are based on autoimmune mechanisms.^[12,13]^ Indirect immunofluorescence, utilizing ethanol-fixed neutrophils as a substrate, is still widely utilized as the primary screening technique for ANCA detection in many clinical laboratories.^[14]^ In line with the 1999 International Consensus on ANCA testing, the practice was to utilize IIFA for ANCA screening, followed by immunoassays for PR3 in cases of positive ANCA results.^[15]^ However, the revised 2017 International Consensus on ANCA testing suggested that high-quality immunoassays should be the primary screening method for patients suspected of having AAV without the need for IIFA.^[15]^ This consensus recommendation does not apply to ANCA testing for the diagnosis of chronic inflammatory bowel diseases, autoimmune hepatitis or drug-induced autoimmunity; hence, the commitment to IIFA persists.^[16]^

The IIFA method has some disadvantages in the detection of ANCA, including the inability to distinguish between p-ANCA patterns in cases of ANCA-associated with vasculitis and nonANCA associated with vasculitis. To solve this issue, a Biochip mosaic model has been formed, consisting of ethanol-fixed granulocytes, formalin-fixed granulocytes, and granulocyte Hep-2 cells.^[17]^ This allows a patient’s sample to be evaluated on 3 different biochips in a single well. The observation of formalin-resistant patterns in formalin-fixed granulocyte biochips is considered favorable for “MPO-associated p-ANCA.”

The inclusion of both Hep-2 cells and granulocyte substrates on the biochip with a mixed pattern helps mitigate potential interactions resulting in false-positive p-ANCA reports due to ANA positivity. However, the observation of any cytoplasmic pattern on Hep-2 cells can lead to false-positive reports for c-ANCA. To further enhance the accuracy of ANCA reporting, a biochip mosaic model consisting of 5 chips, including MPO and PR3, in addition to the 3 biochip versions, is also available.^[18]^

In their 2022 study, Larkey et al^[19]^ investigated the impact of quantitative ANA test positivity using the ELISA method on the p-ANCA IIFA test. They found that patients with a positive ANA test result had an almost 4-fold higher rate of positive p-ANCA interpretation compared with the negative samples. In the same study, they noted a correlation between an increase in ANA titers and an increase in the number of positive p-ANCA results. Similarly, in our study, we observed that samples with a positive ANA test result had ~2.5 times more positive p-ANCA results compared with samples with a negative ANA test result. We found that an increase in ANA titers was significantly associated with an increase in p-ANCA positivity. Therefore, when clinicians evaluate positive p-ANCA results, considering the presence of ANA positivity is crucial.

In our study, all 26 patients with a homogeneous pattern showed positive p-ANCA results, and this association was found to be statistically significant. Deka et al^[11]^ conducted a study in 2022 where they reported positive p-ANCA in 31% of patients with a homogeneous ANA pattern. This finding is consistent with our results. Similarly, in another study, the most commonly observed pattern in conjunction with p-ANCA positivity was the homogeneous pattern.^[19]^ The fact that ANA recognizes nuclear antigens may lead to some ANAs appearing to show a p-ANCA pattern, similar to antiMPO antibodies. This phenomenon could also elucidate why the homogeneous pattern, often linked to the presence of antidouble-stranded DNA antibodies, is more commonly observed as a p-ANCA pattern compared with other ANA patterns.^[20]^

In our study, we observed 86 patients who tested negative for ANA but positive for c-ANCA/p-ANCA. Considering that the inclusion criteria for patients in the study required antiMPO and antiPR3 negativity, the presence of these positive p-ANCA or c-ANCA results can be explained by the development of antibodies against other antigenic proteins in neutrophils, such as elastase, cathepsin G and lactoferrin. In this regard, Schulte-Pelkum et al^[7]^ also highlighted the presence of MPO-independent antigens that contribute to the appearance of the p-ANCA pattern.

The limitations of our study include a relatively small sample size, the absence of tests detecting antigens other than MPO and PR3, and the lack of sufficient clinical information regarding the patients.

In conclusion, ANCA is a highly sensitive and specific biomarker for the diagnosis of AAV. However, the accurate detection of this marker using the correct method plays a crucial role in reaching the correct diagnosis. When reporting a positive ANCA result using the IIFA method, the potential interference of a positive ANA result should always be considered.

## Author contributions

**Conceptualization:** Demet Gür Vural, Büşra Usta.

**Formal analysis:** Yeliz Tanriverdi Çayci, Asuman Birinci.

**Investigation:** Demet Gür Vural, Büşra Usta, Kemal Bilgin.

**Methodology:** Demet Gür Vural, Büşra Usta.

**Project administration:** Demet Gür Vural.

**Software:** Kemal Bilgin.

**Supervision:** Kemal Bilgin.

**Validation:** Demet Gür Vural, Yeliz Tanriverdi Çayci, Asuman Birinci.

**Visualization:** Demet Gür Vural.

**Writing—original draft:** Demet Gür Vural.

**Writing—review & editing:** Yeliz Tanriverdi Çayci, Asuman Birinci.
